# The conscious access hypothesis: Explaining the consciousness

**DOI:** 10.4103/0019-5545.39752

**Published:** 2008

**Authors:** Ravi Prakash

**Affiliations:** Department of Psychiatry, Central Institute of Psychiatry, Ranchi, India

**Keywords:** Computation, conscious access, consciousness, global workspace, information processing, neurobiology, quantum consciousness

## Abstract

The phenomenon of conscious awareness or consciousness is complicated but fascinating. Although this concept has intrigued the mankind since antiquity, exploration of consciousness from scientific perspectives is not very old. Among myriad of theories regarding nature, functions and mechanism of consciousness, off late, cognitive theories have received wider acceptance. One of the most exciting hypotheses in recent times has been the “conscious access hypotheses” based on the “global workspace model of consciousness”. It underscores an important property of consciousness, the global access of information in cerebral cortex. Present article reviews the “conscious access hypothesis” in terms of its theoretical underpinnings as well as experimental supports it has received.

Consciousness represents one of the most complicated issues of nature, which has puzzled philosophers, scientists, mathematicians and the layman alike. Theorists have even gone to the extent saying that, this issue is analogous to other pertinent questions of nature, like origin of the four basic forces of nature and the origin of universe itself.[1–4] No wonder analysts have converged from different fields to apply their ideas to find a clue to this enigma, curiosity being their major driving force. Thanks to the convergence which made the mystery emerge from the fields of abstractness and empiricism and made it an objective scientific topic in spite of its apparent intangibility.

## GRAVITATION AND CONSCIOUSNESS: IS THE HISTORY OF SCIENCE REPEATING ITSELF?

I find the issue very similar to the intractable problem of origin of the gravitational force. The mysterious force of gravitation is likely to resist a complete scientific description in near future. But its journey from the classical Newtonian mechanics to Einstein's space-time curvature, to Hawking's Instanton theories, has definitely been spectacular. It is exciting to see how Einstein's view of gravitation as a space-time curvature got through the scientific community and how “M” theory describes it as an interaction between dimensions of universe.[5,6] With every new theory, newer mystic aspects came into limelight. But the approach remained that of science, involving hypothesis, testing, and implication. Looking back at the history of development of concept of consciousness, we can find a similar evolution, although at a very early stage of course. To start with, earliest records of a description of consciousness as a force (or more accurately as a field phenomenon) can be found in Indian sacred texts. Although the details of ancient concepts are out of the scope of this article, but simply speaking, they were the first to propose a non-material nature of consciousness. The words Chetna and Aatma clearly implied something very close to the entity for which we use consciousness.[7–9] Perhaps that was the origin of the dualistic approach latter echoed in the sacred writings of *Bible* and *Quran* and finally in the Descartean dualistic descriptions. Since that time, philosophical approaches ruled the arena, perhaps because of absence of proper investigational tools and sophisticated branches of science such as cognitive neuroscience and quantum mechanics. Thus, it is quite understandable that the inquisitive minds had to wait till 1940s (when interestingly both events happened simultaneously, i.e., the births of cognitive sciences and quantum mechanics and inventions of PET, SPECT, and fMRI) to begin a true scientific movement. No wonder that the following time was a period of upsurge of interest for using a rational way of studying consciousness. This interest grew exponentially, leading to proposal of new theories, carrying out of novel studies and finally establishment of new departments for consciousness in existing neuroscientific labs and of new centers for consciousness studies in 1980s. The impact of this proliferation of ideas about consciousness can be easily understood by having a look at the magnitude of the number of theories and the amount of work done on the subject, in hardly a decade of this indulgence. It was this indulgence in the past decade, which made to earn the title of “Brain decade”.[10] The impact is definitely going to increase in future, as the effort to go beyond the horizon continues.

## CONSCIOUSNESS IN DIFFERENT FRAMES OF REFERENCE

Conscious experience is not a unitary object, but a set of dimensions. When we experience fear, for example, a very evident conscious state, it is not just a state of unpleasant experience. There are many events happening consecutively in neurophysiology (the dopaminergic outflow in amygdaloid body for example), neurocognition (evaluation of the fear provoking situation, planning for consequent action), body (the perspirations, palpitations, changes in autonomic function), and subjective feeling states (the unpleasantness, increased attentiveness). None of these events can happen without a conscious involvement, in this case, in the form of fear. Therefore, consciousness can be best understood in terms of a set of various dimensions, similar to the space-time dimensions of universe. It seems that it is this dimensional nature of consciousness that has led to adoption of different frames of reference for study and thus various concepts of consciousness. Broadly speaking, there are three major frames of reference prevalent at present, out of which, I have dealt with the cognitive frame later, in details.

### Philosophical frame of reference

Needless to say, this is the oldest approach to consciousness, which still occupies an important position in the community of consciousness logicians. These theories can be supposed to be various combinations of the following three ideas:

1. *The dualism*: This theory is the descendant of the original dualistic separation of mind-body proposed first by Plato in 4th century,[11] then carried ahead by Descartes in 1961.[12] This view of consciousness argues that the neural and experiential properties of consciousness can be correlated but cannot be explanatory one for the other, i.e., they are inherently different entities and the explanatory gap between them cannot be filled. The theory accepts that there can be neural correlates of consciousness (NCC) but they are supposed to be different from the subjective awareness.
2. *The theory of functionalism*: This view proposes that the essence of consciousness lies in the functions it serves, i.e., in a subset of transformations of input and output, which our nervous systems achieve.[13] According to this theory, human consciousness can be understood as an operation of some virtual machine in the parallel architecture of brain. Proponents of this theory have even gone ahead conceptualizing “seeing” as “a way of acting” rather than as the creation of a visual experience.[14]
3. *The identity theory*: This line of argument speaks against the conception of reduction phenomenon and argues that the conscious experience has subjective properties, which are not fully specified by and cannot be reduced to the neural structures or the physical processes to which they are correlated, i.e., it has an altogether different identity. The idea sees consciousness as the main entity and the neural correlates are the ways in which the conscious force uses the brain for serving its purposes.[15,16]

### The neurobiological frame of reference

Bluntly speaking, these theories attempt to correlate any state of awakening with the underlying states of brain activities. Popular as NCC, these are the anatomic, physiological, chemical, and electrical events in neurons, which can be correlated with any state of subjective awareness. Potential candidates have been the reticular activating system (RAS), thalamocortical loops, prefrontal cortex, parietal cortex, neurons of layers three and four of cortex, qEEG correlates of sleep and wakefulness, and so on, the list is exhaustive.[17–20]

Recently, quantum approaches have given birth to many theories. These theories are a special kind of neurobiological theories, which try to explain consciousness on the basis of wave-particle quantum states in neurons, which are supposed to exist in constituent elements of neuron, for example, cytoskeleton.[4]

## UNDERSTANDING COMPUTATION

Before we proceed further to cognitive theories of consciousness, it is important to have a clear idea about what exactly is a computation? Although the term is widely used, it has been found to be used with different connotations in different texts. The original proposition of computation was given by Jerry Fodor, which he said to have derived from Alan Turing:

“Given the methodological commitment to materialism, the question arises, how a machine could be rational?……. Forty years or so ago, the great logician Alan Turing proposed an answer to this question….. Turing noticed that it isn't strictly true that states of mind are the only semantically evaluable material things. The other kind of material thing that is semantically evaluable are symbols…. Having noticed this parallelism between thoughts and symbols, Turing went on to have the following perfectly stunning idea. “I'll bet”, Turing said “that one could build symbol manipulating machine whose changes of state are driven by the material properties of the symbols on which they operate (for example, by their weight or their shape or their electrical conductivity). And I'll bet one could so arrange things that these state changes are rational in the sense that, given a true symbol to play with, the machine will reliable convert it into other symbols that are also true”.[21]

Thus, Fodor sticks to the idea that computation is a symbol manipulation of a neurally realized computer. In simpler words, it is a causal/mechanical process in which language-like representing vehicles are recognized and transformed into a semantically coherent fashion purely on the basis of their syntactic properties.

Thus, symbol manipulation was the main proposal at the time of inception of cognitive neurosciences.

But there is another form of computation, which has not been talked much about and does not have a precise formal definition - the analog computation. Analog computers employ internal models that physically or structurally resemble their representanda. Analog computation is thus not properly conceived as symbol manipulation, but as a physical process driven by the structural properties of analog representational media. This concept receives importance for studying consciousness because one of the major cognitive theories of consciousness (as shall be discussed in details later) relies on this type of computational activity in brain.[22]

## RATIONALE BEHIND COMPUTATIONAL ANALYSIS OF CONSCIOUSNESS

Computation is a marvel of human brain. But why would any one study it for analyzing consciousness, specifically when already there are sophisticated neurobiological theories, which have gone to the extent of exploring the quantum aspects of brain to get clues to the mystery?[23–25,4] The answer lies in the fact that the very birth of this branch of science happened because it was able to give a theory of mind - the computational theory - in which all conscious states had some particular computational correlates. It gave a seat to consciousness in the brain in the form of information processing events. The idea, that many properties of consciousness can be understood if we take into account the information processing going on in some particular conscious act, remains to be as appealing as was in the beginning. Forthcoming years showed an increasing interest in the community of cognitive neuroscientists, which led to a closer look into the theoretical aspect of consciousness, ultimately resulting in the following three encouraging observations:

1. If consciousness has any biological function, it must ultimately be manifested in behavior, which will require information processing by brain.
2. A considerable amount of information processing occurs without conscious involvement, as compared to the few, which happen when we are aware. Thus, what elevates these specific information crunching processes to the political power of consciousness remains a matter of speculation. But surely, these processes force a cognitive neuroscientist to think that they must be intimately related to consciousness and thus candidates for exclusive studies.
3. There are some cognitive tasks such as working-memory acts, spontaneous generation of intentional behavior, and conscious modulation of perceptive computations, which require considerable conscious efforts so that it is impossible to carry out these tasks with out special properties of consciousness.[26,27,16] Thus, it becomes quite easy to assume that these cognitive events represent some important aspect of conscious information processing.

## COGNITIVE THEORIES OF CONSCIOUSNESS

With so much said in the backdrop of cognitive correlates of consciousness, we next come to cognitive theories of consciousness proper. Broadly speaking, there are two categories of theories of consciousness, demarcated perhaps right from the origin of the branch. They are: (i) information processing/computational theories, which use classical symbol manipulation computing and (ii) representational/vehicle theories, which use the language of analog computing.

### Information processing theories of consciousness

These theories seek to explain phenomenal experience in terms of special kinds of computational processes to which some of the brain's representational vehicles are subject. On this view, we become conscious of any representational content in any parallel distributed neuronal network, when their vehicles are able to do some particular computation jobs, independently of any particular intrinsic property of those vehicles. What counts therefore, is “what representational vehicles do rather than what they are”. Typically, the claim is that the content of a representational vehicle is conscious when the vehicle has some privileged computational status, say by being available to an executive system or being inferentially promiscuous.[28] Consciousness is then a result of the rich and widespread informational access relations possessed by a subset of the information bearing states of a cognitive system.[13,29–36]

By far, the most popular among these theories is the one that proposes that consciousness depends on information access, that is, on the ability of the cerebral cortex to be able to access any particular information processing phenomenon, which becomes possible by the virtue of the particular computation process generating the information. This approach will be our topic of further discussion.

### Representational theories of consciousness

On the contrary, these theories propose that consciousness is determined by the presence of some special intrinsic property of representational vehicles themselves, independently of any computations in which those vehicles engage. On this view, consciousness is expected to be explained in terms of the way the information is represented in any neuronal network. Thus, for example, when watching a scene, what it is like for anyone to have the visual experience is to be explained in terms of the way in which the objects in the visual field - their shape, color relative size, and location, etc. - are represented in the individual's brain. In other words, consciousness depends on some aspect of the physical medium of representation.[37–40] It is to be recalled that this way of viewing any cognitive event is similar to and in fact, based on the analog computing phenomena, in which the internal representation of information in the computer is similar structurally to the external representanda.

## THE GLOBAL WORKSPACE MODEL OF CONSCIOUSNESS

This is an information processing theory, which has been amongst the most popular theories of consciousness. Before going into minute details of the theory, I will present a brief history of the concept.

The idea that brain has both conscious and unconscious events going on at the same time is not new. In 1959, the mathematician (and the coiner of the term “artificial intelligence”) John McCarthy, commenting on Oliver Selfridge's pioneering Pandemonium, the first model of a competitive, non-hierarchical computational architecture, clearly demarcated the outline of the global workspace model: “I would like to speak briefly about some of the advantages of the pandemonium model as an actual model of conscious behaviour. In observing a brain, one should make a distinction between that aspect of the behaviour which is made available consciously and the one which is not. If once conceives of the brain as a pandemonium - a collection of demons - perhaps what is going on within the demons can be regarded as the unconscious part of thought and what the demons are publicly shouting for each other to hear, as the conscious part”.[41]

In the classic paper, the psychologist Paul Rozin[42] argued that, “Specializations… form the building blocks for higher level intelligence… and at the time of their origin, these specializations are tightly wired into the functional system they were designed to serve, thus remaining inaccessible to other programs or systems of the brain. I suggest that, in the course of evolution, they become more accessible to other systems and, in the extreme, may rise to the level of consciousness and be applied over the full realm of behavior or mental function (p. 276).”

But these were only theoretical constructs. The first experimental works in the field were done by Newell and Simon in 1972.[43] They were the first to show the utility of a global workspace capacity in a complex system of specialized knowledge sources, which could cooperatively solve problems that no single source could solve alone. But it was not linked to the idea of consciousness then.

The concept that consciousness might be the representative of that global workspace capacity was first presented as a theory by Baars in 1983,[29] when he elaborated, “consciousness is accomplished by a distributed society of specialists that is equipped with working memory, called as ‘global workspace’, whose contents can be broadcast to system as whole”.

In other words, conscious contents provide the nervous system with coherent global information. This theory suggests a correlation of consciousness to a fleeting memory capacity, in which only one consistent content can be dominant at any given moment. The dominant information is widely distributed in the brain, as per the need of the conscious event.

The theory was further evaluated and elaborated by many cognitive neuroscientists with different new emerging themes, the details of which are out of the scope of this article.[1,6,7,44–46]

Overall, all these themes suggested that at any given point, many modular cerebral networks are active in parallel and they keep processing information in an unconscious manner. An information becomes conscious, however, if the neural population that represents it is mobilized by top-down attentional amplification in a brain-scale (i.e., becomes globally available in brain) state of coherent activity that involves many neurons distributed throughout the brain. The long distance connectivity of these “workspace neurons” can, when they are active for a minimal duration, make the information available to a variety of cognitive processes including perceptual categorization, long-term memorization, evaluation, and intentional action. As per the theory, it is this global availability of information through the workspace, what we subjectively experience as a conscious state. Due to this global access of information, it has also been called as “conscious access hypothesis”.

This privilege of globalization of information access does make sense in a nervous system viewed as a massive distributed set of specialized networks. In such a system, events such as coordination, control, and problem-solving could take place by way of a central information exchange, allowing some regions - such as sensory cortex - to distribute information to the whole. This strategy actually works in large-scale computer architectures, which show typical “limited capacity” behavior when information flows by way of a global workspace.[33] A sizable body of evidence suggests that consciousness is the primary agent of such a global access function in humans and other mammals.

## RECENT EVIDENCES FROM VARIOUS STUDIES: LIVING UP TO THE EARLIER EXPECTATIONS

In this section, I will quote evidences from recent studies in favor of earlier predictions made by the global workspace model and will try to correlate it with the concept.

1. *Conscious vs unconscious perception*: With perception, logical implication of the theory was that, conscious perception involves more than sensory analysis. It enables access to widespread brain areas, whereas unconscious input processing is limited to sensory brain regions.
  *Evidence*: Dehaene *et al.*[47] have shown that backward-masked visual words activate mainly visual cortex, whereas identical conscious event evoke widespread visual, parietal, and frontal activation. Tononi and Edelman[48] and Srinivasan *et al.*[49] have demonstrated in binocular rivalry that conscious visual input evokes more intense and coherent MEG responses from flicker-tagged input than matched unconscious stream.
2. Consciousness is needed for comprehension of novel information such as combination of new words. Even common understanding points out that the unconscious input would be processed in a very limited way as compared to conscious input processing. But it was never used to study consciousness. Recent studies have took the point seriously and multiple experiments conducted, replicated the same finding that the outcome of unconscious processing was very little information processing.
  *Evidence*: Repeated efforts to demonstrate multiple word subliminal priming have not succeeded.[50] Likewise, multiple word effects in unattended listening have not been shown to work.[51] Complex unconscious processes do exist in automatic functions and implicit cognition, but unconscious input processing seem to be quite limited.[52]
3. *Consciousness and working memory*: Working memory involves deliberation, which can be technically considered as a form of conscious involvement. It incorporates many cognitive functions such as perceptual input, visual imagery, inner speech, rehearsal, and recall. Thus, by logic, it involves multiple brain functions, which have been substantiated by findings of recent studies.
  *Evidence*: John[53] found that quantitative EEG across six working memory tasks showed three widespread cortical components in the form of gamma band coherence, accounting for 90% of the variance in conscious persons, whereas in anaesthetized nonconscious conditions, there was a loss of gamma band activity in major quadrants of the cortex.
4. *Consciousness and learning*: It seems that all forms of learning involve conscious involvement. Till date, there is no robust evidence that long-term unconscious input of information can lead to its storage. On the other hand, the evidence of learning of conscious events is quite strong. Keeping in view the many information processing events involved in the process of learning and storage of information, especially the learning of novel skills involves much wider areas of cerebral cortex, again giving a validating point for the theory of wider cortical involvement in conscious tasks.
  *Evidence*: Standing in 1973,[54] found that recognition accuracy for 10,000 pictures shown for 5 s each for several few days was better than 96%. This suggests that whatever becomes focally conscious enters episodic memory. Reber in 1989[55] demonstrated through implicit learning paradigms that even implicit learning involves conscious involvement. Haier[56] suggested that novel skill learning activated larger areas of cortex. But after automatcity, the identical tasks tended to involve only restricted regions.
5. *Voluntary control and consciousness*: Like consciousness, voluntary control is a controversial phenomenon. Similar complex phenomenon (cognitive and behavioral) can be made to happen under either voluntary or involuntary control. As voluntary control is a conscious phenomenon, it has also been used as an experimental variable and has been found that, with training, conscious access can be made to any neuronal group or even single neurons, again strengthening the notion that consciousness allows for widespread access in brain.
  *Evidence*: Evans and Abrabanel[57] have shown that conscious feedback training provides with spectacular examples for the scope of the conscious access to almost any neuronal population. Simard and Basmajian[58] have shown that, with auditory feedback, even single motor neurons can come under voluntary control.

## POSSIBLE MECHANISMS OF GLOBAL ACCESS

At present, the brain mechanisms for consciousness remain unclear. Possible neural candidates are:

1. The complex executive neurons in prefrontal cortex.[6]
2. The complexity of reentrant thalamocortical dynamics.[2]
3. NMDA synapses.[59]
4. The gamma asynchrony across cerebral cortex.[46]
5. Thalamocortical distribution from the sensory cortex.[60]

## CONCLUSION

Although defining consciousness is still a major problem, but one of its properties seems to be integration of brain functions, which are otherwise separate and inaccessible neuronal populations. In the light of novel research methods using more direct investigation tools, this property of consciousness becomes even more clear and understandable. No doubt this theory was one of the most propagated and studied in the past “Brain decade”.

In addition, the ease with which it can explain complex cognitive problems, such as working memory and volition, is another reason why this “Global workspace model" should be further explored, as has been worked up on till now.
